# High-Density Lipoprotein Modifications: Causes and Functional Consequences in Type 2 Diabetes Mellitus

**DOI:** 10.3390/cells13131113

**Published:** 2024-06-27

**Authors:** Xiaodi Zhang, Emiel P. C. van der Vorst

**Affiliations:** 1 Institute for Molecular Cardiovascular Research (IMCAR), RWTH Aachen University, 52074 Aachen, Germany; xiazhang@ukaachen.de; 2 Aachen-Maastricht Institute for CardioRenal Disease (AMICARE), RWTH Aachen University, 52074 Aachen, Germany; 3 Interdisciplinary Center for Clinical Research (IZKF), RWTH Aachen University, 52074 Aachen, Germany; 4 Institute for Cardiovascular Prevention (IPEK), Ludwig-Maximilians-University Munich (LMU), 80336 Munich, Germany

**Keywords:** high density lipoprotein, post-translational modifications, HDL dysfunction, type 2 diabetes mellitus, cardiovascular disease

## Abstract

High-density lipoprotein (HDL) is a group of small, dense, and protein-rich lipoproteins that play a role in cholesterol metabolism and various cellular processes. Decreased levels of HDL and HDL dysfunction are commonly observed in individuals with type 2 diabetes mellitus (T2DM), which is also associated with an increased risk for cardiovascular disease (CVD). Due to hyperglycemia, oxidative stress, and inflammation that develop in T2DM, HDL undergoes several post-translational modifications such as glycation, oxidation, and carbamylation, as well as other alterations in its lipid and protein composition. It is increasingly recognized that the generation of HDL modifications in T2DM seems to be the main cause of HDL dysfunction and may in turn influence the development and progression of T2DM and its related cardiovascular complications. This review provides a general introduction to HDL structure and function and summarizes the main modifications of HDL that occur in T2DM. Furthermore, the potential impact of HDL modifications on the pathogenesis of T2DM and CVD, based on the altered interactions between modified HDL and various cell types that are involved in glucose homeostasis and atherosclerotic plaque generation, will be discussed. In addition, some perspectives for future research regarding the T2DM-related HDL modifications are addressed.

## 1. Introduction

Type 2 diabetes mellitus (T2DM) is a major public health problem, affecting nearly 500 million people worldwide [1]. People suffering from T2DM are at high risk of developing microvascular (retinopathy, nephropathy, neuropathy) and macrovascular (peripheral artery, coronary artery, and cerebrovascular) complications [1]. Furthermore, cardiovascular disease (CVD) is recognized as the leading cause of death in T2DM patients [2,3]. A key contributing factor to this cardiovascular risk is diabetic dyslipidemia, which is a cluster of abnormalities in the concentration and composition of plasma lipids and lipoproteins [4]. During the course of T2DM, an increase in plasma triglycerides and low-density lipoprotein (LDL) as well as a decrease in high-density lipoprotein (HDL) levels are commonly observed [5]. Numerous studies have shown that lower levels of HDL cholesterol (HDL-C) are indeed associated with increased risk of CVD [6,7,8]. This reverse relationship is supported by the cardioprotective effects of HDL, such as anti-oxidative, anti-thrombotic, and anti-inflammatory properties in addition to its important role in the reverse cholesterol transport (RCT), in immune process modulation, and in the inhibition of endothelial dysfunction [4,9,10]. Besides the changes in HDL-C levels, HDL dysfunction in relation to structural alterations and metabolic abnormalities of HDL is also correlated with the pathogenesis and prognosis of T2DM [11]. Moreover, a strong inverse association has been shown for HDL cholesterol efflux capacity in macrophages with both subclinical atherosclerosis and obstructive coronary artery disease, independent of the HDL-C levels [12].

Increasing HDL-C concentrations has long been considered as a potential target to reduce cardiovascular risk. However, several clinical trials have shown that increasing HDL-C levels with the use of, for example, niacin or torcetrapib has limited beneficial effects on cardiovascular risk in individuals with or without T2DM [13,14,15,16]. These findings are further supported by a mendelian randomization study, which showed that specifically raising plasma HDL-C by genetic mechanisms does not lower the risk for a myocardial infarction [17]. It is therefore suggested that improving HDL function, rather than simply raising cholesterol levels, deserves more attention in the treatment of T2DM and its complications [18]. Due to the persistently elevated concentrations of blood glucose and long-term inflammatory conditions in T2DM, HDL undergoes post-translational modifications (PTMs) such as oxidation, glycation, and carbamylation [18,19,20]. Modifications in the HDL lipidome and proteome affect the HDL composition and are thought to be potential molecular mechanisms of HDL dysfunction [21,22]. However, it remains less clear whether and how modifications of HDL are involved in the pathogenesis of T2DM. Therefore, this review explores how T2DM affects modifications of HDL particles and how HDL modifications may contribute to the development of T2DM as well as T2DM-related complications.

## 2. HDL Structure and Function

### 2.1. HDL Structure

HDL particles are a group of small, dense, and protein-rich lipoproteins, with a mean size of 8–10 nm in diameter and a density ranging from 1.063 to 1.21 g/mL [23]. Mature HDL particles have a micelle-like configuration, where the shell contains mainly phospholipids and unesterified cholesterol forming a surface monolayer, with a core consisting of cholesteryl esters and triglycerides [24]. In addition to lipids, HDL also contains a large number of proteins including apolipoproteins, lipid transfer proteins, enzymes, acute-phase response proteins, and other protein components. The most abundant HDL protein is apolipoprotein (apo) A-I (~70%), followed by apoA-II (~15–20%), which are major functional apolipoproteins and contribute to the stability of HDL [25]. ApoA-I and apoA-II have been shown to play different roles in HDL metabolism, as apoA-I mainly facilitates cholesterol efflux whereas apoA-II reduces scavenger receptor class B type I (SR-BI)-mediated cholesterol uptake by the liver [26]. Most HDL particles contain both apoA-I and apoA-II. However, about 10% of HDL particles contain only apoA-I without apoA-II, which has distinct functional properties [27]. HDL apolipoproteins are embedded in the phospholipid surface of HDL with an amphipathic α-helical structure, in which the hydrophobic face drives the association with lipids and the hydrophilic face confers water solubility of the HDL particles [25]. Other HDL apolipoproteins include apoA-IV, apoCs, apoD, apoE, apoF, apoH, apoJ, apoL-I, and apoM [23].

Due to the diverse compositional features, HDL particles are highly heterogeneous in density, size, shape, composition, and hence also in physiological function. According to these differences, HDL particles can be divided into various subfractions, which are classified based on the used separation methodology (Table 1). The gold standard technique, analytic ultracentrifugation, allows the separation of the two main HDL subclasses: the larger, less dense (1.063–1.125 g/mL), and relatively lipid-rich HDL2 particles as well as the smaller, denser (1.125–1.21 g/mL), and relatively protein-rich HDL3 particles [26,28,29]. Using non-denaturing polyacrylamide gradient gel electrophoresis, based on particle size, HDL2 and HDL3 can be further separated into HDL2b (9.7–12.0 nm diameter) and HDL2a (8.8–9.7 nm), as well as HDL3a (8.2–8.8 nm), HDL3b (7.8–8.2 nm), and HDL3c (7.2–7.8 nm) [30,31]. In recent years, nuclear magnetic resonance spectroscopy is increasingly used for the identification and quantification of HDL subclasses based on particle size: large HDL (8.8–13.0 nm), medium HDL (8.2–8.8 nm), and small HDL (7.3–8.2 nm) [23]. Moreover, agarose gel electrophoresis separates HDL according to surface charge and shape, resulting in the identification of spherical α-HDL and discoidal preβ-HDL. In combination with a 2-dimensional electrophoretic method, these two HDL subclasses can be further separated according to their mobility and size: preβ- (preβ1 and preβ2), α- (α1, α2, α3 and α4), and preα- (preα1, preα2, preα3) HDL [32].

**Figure 1:**
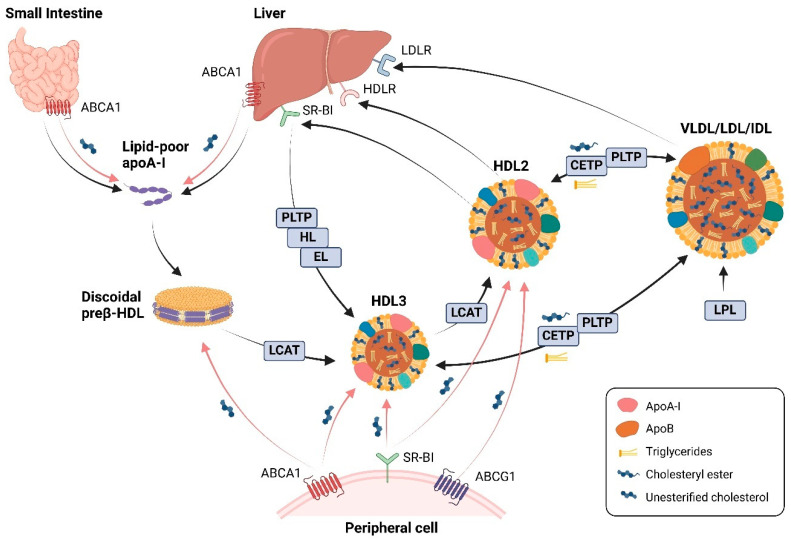
Schematic overview of HDL biogenesis and metabolism. Lipid-poor apoA-I is synthesized in the liver and the small intestine. Following the secretion, apoA-I interacts with the ATP-binding cassette (ABC) transporter ABCA1, forming discoidal preβ-HDL particles. Continued efflux of unesterified cholesterol via ABCA1, ABCG1, or scavenger receptor class B type I (SR-BI) from peripheral cells and subsequent cholesterol esterification via lecithin:cholesterol acyltransferase (LCAT) lead to the conversion of discoidal HDL into HDL3 and subsequently into HDL2. After traveling in the circulation, the cholesterol is transported to the liver for excretion, either directly by SR-BI-mediated uptake, or indirectly after transfer to apoB-containing lipoproteins such as very low/low/intermediate density lipoprotein (VLDL/LDL/IDL) by cholesteryl ester transfer protein (CETP), followed by the uptake of LDLs by the LDL receptor (LDLR) in the liver. Furthermore, lipoprotein lipase (LPL) catalyzes the VLDL hydrolysis and phospholipid transfer protein (PLTP) transfers phospholipids from LDLs to HDL, thereby promoting HDL maturation. HDL2 can be remodeled and form lipid-poor HDL, catalyzed by plasma PLTP, hepatic lipase (HL), and endothelial lipase, for further lipidation. Additionally, HDL can be catabolized in the liver via an unidentified HDL receptor (HDLR). Created with BioRender.com.

The biogenesis of HDL is a complex process that begins with the secretion of lipid-poor apoA-I by the liver (~80%) and the small intestine (~20%) [33]. The secreted apoA-I are subsequently lipidated on the surface of hepatocytes and enterocytes via interaction with the ATP-binding cassette (ABC) transporter ABCA1, which enables the transfer of cellular phospholipids and unesterified cholesterol to lipid-poor apoA-I, leading to the formation of discoidal preβ-HDL particles [34,35]. Subsequent esterification of free cholesterol by the plasma enzyme lecithin:cholesterol acyltransferase (LCAT) converts the discoidal particles to spherical HDL particles [36]. Moreover, LCAT-mediated esterification of newly acquired free cholesterol in small spherical HDL can further lead to generation of large HDL particles (Figure 1). During these processes, cellular cholesterol efflux to small, dense HDL particles is mainly mediated by ABCA1, whereas large HDL (primarily HDL2)-induced cholesterol efflux is frequently related to ABCG1 [25,37]. However, ABCG1-mediated efflux does not appear to impact steady-state HDL-C levels [38]. Additionally, in some cells such as macrophages, SR-BI has been shown to also contribute to the efflux to both HDL2 and HDL3 [36]. Eventually, the cholesterol is transported to the liver for clearance, either directly by selective uptake through SR-BI or indirectly after transfer to apoB-containing lipoproteins such as LDL in exchange for triglycerides by cholesteryl ester transfer protein (CETP) [24,39]. Furthermore, lipoprotein lipase (LPL) catalyzes the hydrolysis of triglycerides which are present in very low-density lipoproteins (VLDL), and phospholipid transfer protein (PLTP) transfers phospholipids from these apoB-containing lipoproteins to HDL, thereby facilitating apoB-containing lipoprotein catabolism and HDL maturation [40,41]. Spherical HDL can be remodeled in the circulation under the catalysis of plasma PLTP [42], hepatic lipase [43], and endothelial lipase [44], and it can be further catabolized in the liver via an unidentified HDL receptor-dependent uptake [45] (Figure 1).

### 2.2. HDL Function

Distinct subclasses of HDL often contain particular proteins with similar or complementary functions, which play a crucial role in the functionality of HDL such as its protease inhibition, lipid metabolism and transport, hemostasis, anti-oxidant, or anti-inflammatory properties [46]. These properties of HDL protect organisms from chemical or biological damage, or help tissue repair, linking to cardiovascular protection. The underlying mechanisms mainly involve cholesterol transport, interaction with cells, as well as detoxification of hazardous molecules [24].

#### 2.2.1. Reverse Cholesterol Transport

The major mechanism of HDL-mediated cardioprotection is considered to be the RCT, the pathway by which excess cholesterol from peripheral cells (including macrophages in the arterial wall) is acquired by HDLs and transported to the liver for excretion. As described above, HDL induces cholesterol efflux via either aqueous diffusion and facilitated diffusion mediated by SR-BI, or active transportation with ABCA1 and ABCG1 [47] (Figure 1). It has been shown that such processes protect cells against accumulation of intracellular free cholesterol and simultaneously also contribute to regulations of several cellular responses [24].

#### 2.2.2. Cellular Interactions

HDL can interact with various cell types, for example, immune cells, endothelial cells, pancreatic beta cells, adipocytes, and myocytes and thereby regulate inflammation, angiogenesis, and glycemic control [48,49,50,51,52]. HDL particles or HDL components regulate cellular responses mainly via alterations of cholesterol homeostasis as well as interaction with specific receptors on the cell surface followed by induction of intracellular signaling [53].

HDL-driven RCT is of particular importance to remove excess cholesterol from macrophages and thereby prevent accumulation of cellular cholesteryl ester and formation of macrophage foam cells, which are hallmarks of early atherosclerotic lesions [54]. Cellular cholesterol levels have been linked to the modulation of macrophage functions [55]. Additionally, there are also studies suggesting that HDL may regulate inflammatory responses in macrophages independently of intracellular cholesterol level changes, although these effects remain less straightforward [56,57,58]. It has on the one hand been shown that HDL can protect bone marrow-derived macrophages (BMDMs) from toll-like receptor (TLR)-induced inflammation by inducing the transcriptional regulator *Atf3* [56] and downregulate the type I interferon (IFN) receptor signaling [57]. On the other hand, there are also findings showing that HDL can promote inflammation in both BMDMs and human primary macrophages via activating the protein kinase C (PKC)-NF-κB-STAT1-IRF1 signaling, which seems to be mediated by passive cholesterol depletion from the macrophage cell membrane [58]. Deficiency of ABCA1/ABCG1/SR-BI did not affect this pro-inflammatory effect of HDL in macrophages [58]. These contradictory findings might be explained by the different experimental conditions and different HDL preparations [59]. Despite these discrepancies, the pro-inflammatory effect of HDL seems to be unique for macrophages. Stimulation of HDL-dependent RCT dampens T cell priming and is considered protective in autoimmune diseases [60]. In adipocytes, HDL and apoA-I also inhibit inflammation via removal of cholesterol from lipid rafts regulated by ABCA1, ABCG1, and SR-BI [61]. In addition to inflammation-related regulations, HDL has also the ability to improve cell survival and functionality. HDL protects endothelial cells by interacting with SR-BI and activating endothelial nitric oxide synthase (eNOS) [62], which links to endothelial apoptosis. HDL can also transfer oxidation-prone lipids from LDL and biological membranes for catabolism and prevent oxidized LDL-mediated endothelial dysfunction [63]. Moreover, HDL-associated apoM/sphingosine-1 phosphate (S1P) is shown to interact with S1P receptors and improves endothelial cell function by the regulation of adhesion molecule expression and thereby leukocyte-endothelial adhesion, as well as maintenance of the endothelial barrier integrity [64]. Furthermore, apoA-I also improves functionality of pancreatic beta cell and myocytes and thereby improves glycemic control. In beta cells, apoA-I interacts with ABCA1 and promotes insulin synthesis and secretion via a PKA (protein kinase A)-FoxO1 (forkhead box protein O1)-dependent mechanism [52]. In myocytes, apoA-I is able to stimulate glucose uptake by activation of AMP-activated protein kinase [65].

#### 2.2.3. Detoxification of Hazardous Molecules

In addition to the cellular interactions, the amphipathic property and reverse transport capacity of HDL particles allow them to directly bind and eliminate various hazardous molecules, such as bacterial lipopolysaccharides (LPS), oxidized lipids, and lipophilic xenobiotics [24,66]. It is suggested that HDL facilitates elimination of circulating hazardous molecules either via reverse transport to the liver for clearance or by direct inactivation on the surface of HDL [24]. Lipid transporter proteins PLTP and CETP promote transferring and binding of LPS to HDL [67], and subsequently LPS can be neutralized by direct interaction with apoA-I [68]. Similarly, HDL also has the ability to neutralize DNA/RNA viruses. Besides neutralizing, HDL can block several viruses from entering cells by competitive interaction with SR-BI [66]. Moreover, HDL-related enzymes such as paraoxonase 1 (PON1), LCAT, as well as lipoprotein-associated phospholipase A2 are shown to contribute to hydrolysis of oxidized phospholipids [24].

The above findings support that HDL functions are highly heterogeneous and seem to be specific proteome dependent. HDL exerts protective effects on various cell types mainly by regulating inflammation, cell survival, and function. The reverse transport property of HDL is involved in most of those processes, and therefore RCT-related HDL proteome might drive the protective functions of HDL.

## 3. Generation of HDL Modifications in T2DM

Structural and functional changes of HDL have been reported in people with T2DM. Hyperglycemia, oxidative stress, and inflammation that occur during T2DM are thought to be the key contributing factors [69], which can induce various HDL modifications including the PTMs of the protein components and other alterations in the protein and lipid composition of HDL. Here, the main modifications of HDL and their causes in T2DM will be discussed.

### 3.1. Post-Translational Modifications

#### 3.1.1. Glycation

Glycation is a non-enzymatic reaction that occurs when glucose molecules bind to the amino groups of amino acids, peptides, and proteins [70]. In T2DM, persistent hyperglycemia accelerates protein glycation, leading to increased formation of advanced glycation end products (AGEs), which have been closely associated with the development of cardiovascular complications [70,71]. It has been shown that HDL from T2DM patients has a 250% higher level of glycation compared to that from healthy individuals [72]. Plasma HDL that has been glycated by dicarbonyl modification accounts for 4.5% of total HDL in individuals with T2DM, while in healthy controls this glycated HDL makes up 2.6% of the total HDL [73]. These glycation modifications of HDL are mostly located on apoA-I, preferably at lysine or arginine residues, depending on the glycating agents [74,75,76]. For example, modification of HDL/apoA-I by the glucose metabolite methylglyoxal occurs on the arginine amino acids of apoA-I at R27, R61, R116, R123, R131, R149, R151, and R153 [73,75]. Moreover, increased glycation is also observed in HDL-related enzymes such as PON1, LCAT, and CETP in T2DM [74]. Several in vivo/in vitro studies have demonstrated that diabetic glycation of apoA-I as well as these HDL-associated enzymes can result in a reduction in their enzymatic activity and impair HDL function [77,78,79,80,81].

#### 3.1.2. Oxidation

Elevated oxidative stress in T2DM leads to the oxidative modification of HDL and contributes to HDL dysfunction [69]. Myeloperoxidase (MPO), a heme enzyme secreted by macrophages in inflammatory states, is an important source of oxidative stress in the human artery wall and causes HDL oxidation [82]. Increased levels of serum MPO are indeed observed in T2DM individuals and are associated with coronary atherosclerotic plaque progression [83,84]. ApoA-I has been reported to be a selective target for MPO-catalyzed modifications in serum from individuals with T2DM and CVD [85,86]. MPO converts hydrogen peroxide to hypochlorous acid to execute oxidative reactions and modifies apoA-I at the tyrosine and methionine residues [82]. Moreover, MPO also modifies lipids of HDL in T2DM, resulting in increased formation of malondialdehyde as a consequence of HDL lipid peroxidation [87]. A recent study demonstrates that other oxidized lipids in HDL such as oxidized fatty acids are also elevated in T2DM patients and impair the anti-oxidative activity of HDL [88]. Abnormalities such as hyperglycemia and inflammation both promote oxidative stress in T2DM [89,90] and are therefore also potential contributors to the oxidative modifications in HDL. It is found that oxidized HDL is positively correlated with plasma glucose levels in dyslipidemic nondiabetic individuals [91]. A cross-sectional study shows that in T2DM apoA-I oxidation is not associated with glycated hemoglobin, which represents three-month average blood sugar level, but is strongly associated with the duration of T2DM [92]. These results again suggest that regulation of HDL oxidation in T2DM is dependent on a complex combination of several metabolic disorders rather than hyperglycemia dominance.

#### 3.1.3. Carbamylation

Carbamylation is a non-enzymatic irreversible PTM that corresponds to the binding of isocyanic acid to the amino groups of proteins [93]. Carbamylation modification occurs preferentially on the e-NH_2_ of lysine residues, resulting in the formation of carbamyl-lysine (CBL) [94]. In T2DM patients, the CBL content in HDL is doubled in comparison to healthy controls and is positively correlated with MPO concentrations [95], raising the suggestion that the elevated MPO may drive the generation of HDL carbamylation in T2DM. A more recent study indeed demonstrated that MPO induces carbamylation of apoA-I in human aortic atheroma and that this modification mainly occurs on the lipid-poor apoA-I forms [96]. It is also evidenced that at sites of inflammation, MPO-derived chlorinating species induce cyanate formation through decomposition of the plasma components thiocyanate and thereby promote carbamylation of the targeted (lipo)proteins [97]. Carbamylation of HDL causes impairment of the anti-inflammatory and anti-oxidative activities of HDL and is therefore considered a potential mechanism of HDL dysfunction [97].

### 3.2. Other Modifications in Lipid and Protein

Besides the PTMs of HDL, several other alterations in the lipid and protein composition of HDL are also observed in T2DM. It has been revealed that circulating triglycerides and small HDL particles are enriched in T2DM, accompanied by a loss of large HDL particles [98,99]. These shifts in HDL subspecies may be attributed to an abnormal generation and conversion of HDL. It is evidenced that T2DM individuals have higher plasma CETP concentrations compared to healthy controls [100]. Larger HDL2 particles can be converted into smaller HDL3 particles by CETP-mediated exchange of cholesteryl esters from HDL with triglycerides of apoB-containing lipoproteins [101]. It is also demonstrated that the triglyceride-rich and cholesterol ester-depleted HDL particles are usually less stable with loosely bound apoA-I, and are more easily lipolyzed by hepatic lipase, which further results in a reduction in the particle size and loss of apoA-I [102]. In line with this, reduced plasma concentration of apoA-I is indeed observed in T2DM compared to healthy subjects [103]. Moreover, low plasma levels of apoM and its natural ligand S1P [104,105] as well as high levels of apoE [106] are also present in T2DM. ApoM is expressed mainly in the liver and kidneys and exhibits anti-atherogenic effects [107]. Systemic inflammation, as an accompanying condition in T2DM, decreases apoM mRNA levels in the liver and kidney expression, resulting in low circulating levels of apoM [108]. Elevated levels of HDL-bound apoE in T2DM are shown to positively associate with its production rates as well as HbA1c levels, suggesting a potential role of hyperglycemia in the regulation of HDL-bound apoE kinetics [109]. Additionally, depletion of PON1-rich HDL3 particles and reduction in PON1 activity in the serum are also reported in T2DM [110,111], linking to impairment in the anti-oxidative properties of HDL. Apart from the changes in these original components of HDL, T2DM induces an increased binding of the apolipoproteins serum amyloid A (SAA) to HDL particles, resulting in compositional changes and dysfunction of HDL [112]. SAA is an acute phase protein that is synthesized primarily in the liver and secreted in response to inflammation. It has been previously shown that SAA has the ability to directly replace important proteins in HDL such as apoA-I and PON1 [21]. Therefore, it can be hypothesized that an increased presence of SAA in HDL in T2DM may cause a further reduction and disability of these proteins in HDL.

Taken together, these findings suggest that hyperglycemia, inflammation, and oxidative stress in T2DM induce the generation of various modifications to HDL including PTMs of proteins as well as direct changes in lipid and protein compositions of HDL. These modifications lead to HDL dysfunction either via directly impairing the ability of their targeted proteome/lipidome or via complex interactions and ultimately causing extensive functional impairments.

## 4. Effects of HDL Modifications on T2DM

**Figure 2:**
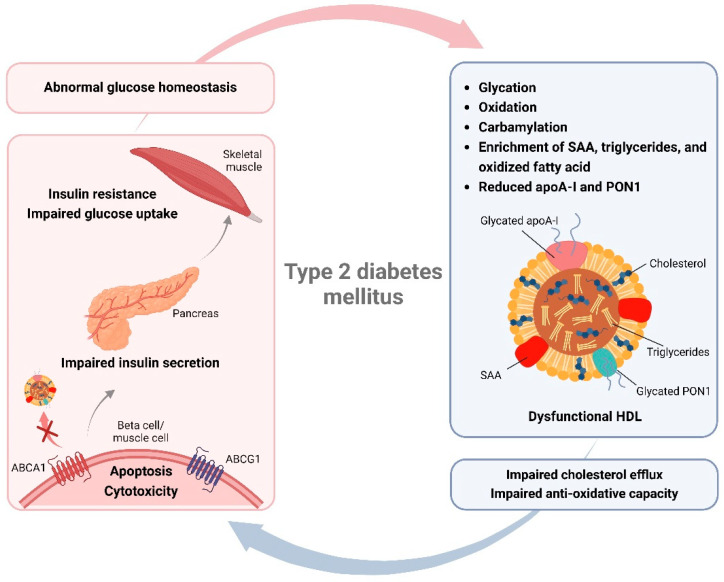
Causes and functional consequences of HDL modifications in type 2 diabetes mellitus (T2DM). Metabolic disorders in T2DM cause the generation and accumulation of various harmful modifications of HDL, which impair cholesterol efflux and anti-oxidative capacities of HDL. In turn, dysfunctional HDL impairs the insulin secretory capacity of pancreatic beta cells, as well as the insulin sensitivity and glucose uptake of skeletal muscles, which consequently disturb glucose homeostasis in T2DM, leading to a vicious cycle. SAA, serum amyloid A; PON1, paraoxonase 1. Created with BioRender.com.

Since the generation of HDL modifications in T2DM impairs the function of HDL and its related proteins, it is believed that these compositional changes in HDL may in turn also impact T2DM progression. T2DM develops when beta cells become dysfunctional and are therefore unable to produce or secrete sufficient insulin, or when insulin-sensitive tissues become insulin resistant and loss their ability to respond adequately to normal levels of insulin. Dysfunctional HDL can promote T2DM by affecting both the insulin secretory capacity of pancreatic beta cells and the insulin sensitivity and glucose uptake of skeletal muscles [11] (Figure 2). A recent study demonstrated that deficiency in apoA-I in mice leads to reduced HDL-C levels and to HDL particles that mainly contain apoE and apoC-II [113]. Moreover, apoA-I deficiency disturbs plasma glucose homeostasis in mice, shown as a reduced glucose tolerance and insulin sensitivity as well as elevated fasting glucose levels, resulting from impaired insulin secretion in beta cells and insulin-resistant skeletal muscles [113]. In T2DM, the stability and plasma half-life of apoA-I and HDL are reduced upon glycation [73,114]. It is evidenced that the extent of glycation in HDL is negatively correlated with plasma HDL-C concentrations [73]. Glycation modifications of apoA-I, especially the methylglyoxal-induced glycation, largely reduces the capability of apoA-I to promote glucose tolerance in an insulin-resistant mouse model [75]. In line with these findings, glycated apoA-I loses the capacity to stimulate glucose uptake in cultured skeletal muscle myotubes and insulin secretion in cultured beta cells [75].

A possible pathway that mediates these HDL modification-induced abnormalities in glycemic control may be the impaired cholesterol efflux, which is also frequently observed in T2DM individuals [115,116]. Under healthy conditions, cholesterol efflux protects beta cells from cholesterol accumulation and thereby facilitates insulin secretion [117]. It has previously been shown that excess cholesterol in beta cells decreases glucokinase activity because of increased neuronal nitric oxide synthase dimerization, leading to decreased insulin secretion and glucose metabolism [118]. AGEs are shown to inhibit both SR-BI-mediated uptake of HDL cholesterol esters as well as cholesterol efflux [119]. Similarly, glycation also destabilizes ABCA1 and reduces its binding to apoA-I, resulting in diminished RCT from cells to apoA-I [120]. Deficiency in ABCA1 in mice causes islet cholesterol accumulation, which is associated with dysfunction of beta cells [121]. In addition to beta cells, downregulation of ABCA1 in muscle fibers also increases cholesterol levels and results in decreased glucose uptake as well as reduced insulin-dependent Akt phosphorylation and GLUT4 translocation, which have been considered as the origin of insulin resistance [122]. Moreover, glycation also affects the levels or activity of other HDL-related enzymes such as CETP and LCAT, which may together contribute to the impaired RCT in T2DM [77,123,124]. Deficiency in LCAT itself, however, does not directly affect the function of beta cells or skeletal muscles in mice [113].

Apart from the RCT, an impaired anti-oxidative capacity of HDL particles also seems to disturb glucose homeostasis in T2DM [11,117]. Under conditions of hyperglycemia and oxidative stress, depletion of apoA-I and PON1, reduction in PON1 activity, and enrichment of oxidized fatty acids in HDL all lead to an impairment of the anti-oxidative capacity of HDL [88,125,126,127]. It has been shown that deficiency in the antioxidant PON1 in mice significantly increases oxidative stress, resulting in impaired glucose tolerance, enhanced insulin resistance, and reduced glucose uptake in skeletal muscles, potentially via inhibition of the insulin receptor substrate 1 (IRS-1)/PI3K/Akt signaling pathway [128]. PON1 addition to cultured muscle cells reverses these effects and promotes GLUT4 expression, thereby improving glucose regulation [128]. Additionally, PON1 can also improve beta cell survival under high glucose conditions and stimulate insulin release [129]. Cell-permeable PEP-1-PON1 fusion protein protects beta cells against cytokine-induced cytotoxicity and restores insulin secretion by reducing oxidative/nitrosative stress, apoptosis, and inflammation [130]. Though not directly proven, these data suggest that HDL modification-related PON1 abnormality could damage skeletal muscle and beta cell function due to enhanced oxidative stress as well as stress-related cell apoptosis or inflammation, thereby further disturbing the glucose homeostasis.

Current findings support that modifications of HDL and its related enzymes in T2DM could further promote the development of T2DM via affecting cell survival and functionalities of both pancreatic beta cells and skeletal muscles. HDL dysfunction, especially impaired cholesterol efflux and anti-oxidative activity of HDL, appear to play a leading role in abnormal glycemic control caused by HDL modifications.

Structural and functional changes in HDL have been reported in people with T2DM. Hyperglycemia, oxidative stress, and inflammation that occur during T2DM are thought to be the key contributing factors [69], which can induce various HDL modifications including the PTMs of the protein components and other alterations in the protein and lipid composition of HDL. Here, the main modifications of HDL and their causes in T2DM will be discussed.

## 5. Effects of HDL Modifications on T2DM Cardiovascular Complications

**Figure 3:**
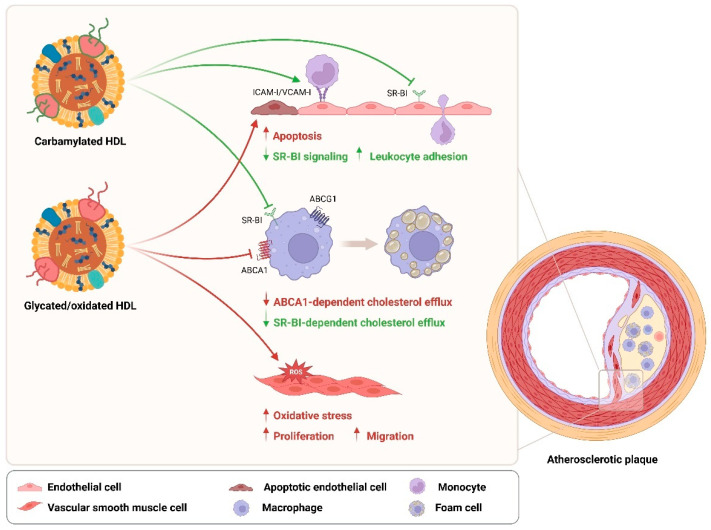
Impact of post-translational HDL modification on atherogenesis. Post-translational modifications of HDL in type 2 diabetes mellitus potentially facilitate the pathogenesis of atherosclerosis, via the disturbance of macrophage cholesterol homeostasis, endothelial functionality, and vascular smooth muscle cell behavior. Abbreviations: ICAM-I, intercellular cell adhesion molecule I; VCAM-I, vascular cell adhesion molecule I; SR-BI, scavenger receptor class B type I; ABCA1, ATP-binding cassette subfamily A member 1; ABCG1: ATP-binding cassette subfamily G member 1. Created with BioRender.com.

HDL has been shown to protect against atherogenesis by stimulating the cholesterol efflux from macrophages, ameliorating endothelial dysfunction, and inhibiting vascular smooth muscle cell (VSMC) proliferation and migration, as well as other anti-inflammatory, anti-oxidative, and anti-apoptotic effects [125,131,132]. Modifications of HDL in T2DM lead to a loss of its anti-atherogenic properties and are therefore considered to be potential risk factors for developing cardiovascular complications. It has been confirmed that serum levels of glycated apoA-I in T2DM patients are positively associated with the severity of coronary artery disease [133]. Similarly, carbamylated HDL serum levels are independently associated with all-cause mortality and cardiovascular-related mortality in T2DM patients [19]. Although the direct association between T2DM-related HDL oxidation and CVD risk remains less clear, MPO-induced oxidation of apoA-I has been implicated in human atherosclerotic lesions [134]. Based on current literature, HDL modifications in T2DM seem to impact the pathogenesis of CVD mainly by altering cholesterol efflux in macrophages, endothelial function, and VSMC proliferation and migration (Figure 3).

Modifications such as glycation, oxidation, and carbamylation are shown to compromise the ability of HDL/apoA-I to efflux cholesterol from macrophages [75,135,136,137,138], which largely increase the risk for the formation of foam cells and thereby contribute to atherosclerotic plaque generation [139]. Impaired cholesterol efflux in J774 macrophages induced by methylglyoxal-modified apoA-I is mediated by the ABCA1 pathway [75]. Besides glycation, AGEs also participate in the alteration of apoA-I-mediated RCT by significantly reducing protein levels of ABCA1 in macrophages, while ABCG1 and SR-BI levels remained unchanged [140]. Similarly, although not tested in macrophages, MPO-induced oxidation of apoA-I impairs its ability to stimulate the Janus kinase 2 (JAK2) signaling and impairs its binding to ABCA1 in baby hamster kidney cells [141]. Interestingly, in contrast, carbamylated HDL affects only the SR-BI-dependent cholesterol efflux in THP-1 macrophages and induces net cholesterol accumulation as well as SR-BI-dependent lipid droplet formation in these cells, while the ABCA1-mediaited cholesterol efflux remains unaffected [135].

In addition to macrophages, carbamylated HDL is also reported to inhibit SR-BI signaling in human aortic endothelial cells (HAECs), accompanied by a suppressed expression of vascular endothelial growth factor receptor-2 (VEGFR2) and impaired endothelial repair capabilities, which can together contribute to endothelial dysfunction [142]. Moreover, carbamylated HDL increases monocyte-to-endothelium adhesion and upregulates the expression of cell adhesion molecules ICAM-I and VCAM-I in human umbilical vein endothelial cells via NF-κB/p65 signal activation [95]. While the impact of glycated HDL on endothelial function seems to be limited, an additional oxidative modification can lead to apoptosis of HAECs via mitochondrial dysfunction [143]. In line with these findings, glycated oxidized HDL also attenuates the expression of eNOS and subsequently decreases the production of NO in HAECs, which can cause a failure of endothelial cells to protect themselves from apoptosis [144].

In VSMCs, oxidative modification of HDL induces activation of NADPH and the production of reactive oxygen species (ROS) and consequently promotes their proliferation and migration [145]. Similar findings are also shown for in vitro glycated HDL or HDL isolated from T2DM patients, which are suggested to trigger VSMC proliferation and migration by raising oxidative stress [146]. These data again support an increased risk for developing atherosclerosis in diabetic subjects.

The above findings suggest that HDL modifications in T2DM can promote the development and progression of cardiovascular complications by affecting the behavior or function of macrophages, endothelial cells, and VSMCs, due to the impaired functions of HDL including its RCT, anti-inflammatory, anti-oxidative, and anti-apoptotic properties.

## 6. Conclusions and Perspective

Diverse compositional characteristics render HDL particles heterogeneous in physiological functions, linking to diabetic and cardiovascular protection. Hyperglycemia as well as enhanced oxidative stress and inflammation in T2DM lead to the formation and accumulation of various harmful modifications of HDL, which are key factors for HDL dysfunction. In turn, T2DM-related HDL modifications impair the insulin secretory capacity of pancreatic beta cells, as well as the insulin sensitivity and glucose uptake of skeletal muscles, which consequently disturb glucose homeostasis in T2DM, leading to a vicious cycle (Figure 2). Additionally, modified HDL in T2DM also displays a high potential to facilitate the pathogenesis of atherosclerosis, possibly via the disturbance of macrophage cholesterol homeostasis, endothelial functionality, and VSMC behavior (Figure 3). However, more investigations are still needed to sufficiently understand the underlying mechanisms behind these complex interactions between HDL modifications and different cell types. It becomes increasingly clear that the pathological consequences of dysfunctional HDL can be modification and cell type dependent. Not all modifications of HDL components show a direct causal link to the development and progression of T2DM or its related cardiovascular complications. A point of concern is that an interrelation commonly exists between disturbed HDL proteome and lipidome. However, how exactly they interact especially during these complex metabolic disorders remains to be studied in the future. Moreover, it has been implied that a single modification to HDL might not sufficiently affect cell function under certain conditions, but the effect could be potentiated by addition of another modification. Since the in vivo pathological conditions often involve multiple modifications simultaneously, single HDL modifications that show no direct effect on cell action in vitro could still be a potential risk factor and should not be ignored. More research is needed also to explore the potential risks of such HDL modifications, particularly in combination with other modifications.

Due to the broad involvement of HDL modifications in the regulation of various T2DM- and CVD-related cells, a better understanding of their potential in the treatment and prevention of cardiometabolic diseases remains essential and a big challenge for future studies.

## Figures and Tables

**Table 1:** Major HDL subclasses according to different separation techniques.

Technique	Measurement	Subfraction
Ultracentrifugation	Density	HDL2 (1.063–1.125 g/mL) HDL3 (1.125–1.21 g/mL)
Non-denaturing polyacrylamide gradient gel electrophoresis	Size	HDL2b (9.7–12.0 nm) HDL2a (8.8–9.7 nm) HDL3a (8.2–8.8 nm) HDL3b (7.8–8.2 nm) HDL3c (7.2–7.8 nm)
Nuclear magnetic resonance	Size	Large HDL (8.8–13.0 nm) Medium HDL (8.2–8.8 nm) Small HDL (7.3–8.2 nm)
Agarose gel electrophoresis	Charge and shape	α-HDL (spherical) preβ-HDL (discoidal)
2-dimensional electrophoresis	Charge and size	preβ-HDL (preβ1 and preβ2) α-HDL (α1, α2, α3 and α4) preα-HDL (preα1, preα2, preα3)
